# Midostaurin after allogeneic stem cell transplant in patients with *FLT3*-internal tandem duplication-positive acute myeloid leukemia

**DOI:** 10.1038/s41409-020-01153-1

**Published:** 2020-12-07

**Authors:** Richard T. Maziarz, Mark Levis, Mrinal M. Patnaik, Bart L. Scott, Sanjay R. Mohan, Abhinav Deol, Scott D. Rowley, Dennis D. H. Kim, Daniela Hernandez, Trivikram Rajkhowa, Kelly Haines, Gaetano Bonifacio, Patrice Rine, Das Purkayastha, Hugo F. Fernandez

**Affiliations:** 1 Oregon Health & Science University, Portland, OR USA; 2 Sidney Kimmel Comprehensive Cancer Center at Johns Hopkins, Baltimore, MD USA; 3 Mayo Clinic, Rochester, MN USA; 4 Fred Hutchinson Cancer Research Center, Seattle, WA USA; 5 Vanderbilt-Ingram Cancer Center, Nashville, TN USA; 6 Karmanos Cancer Institute, Detroit, MI USA; 7 Hackensack University Medical Center, Hackensack, NJ USA; 8 Princess Margaret Cancer Centre, University of Toronto, Toronto, ON Canada; 9 Novartis Pharmaceuticals Corporation, East Hanover, NJ USA; 10 Moffitt Cancer Center, Tampa, FL USA

**Keywords:** Acute myeloid leukaemia, Cancer

## Abstract

We evaluated standard-of-care (SOC) treatment with or without midostaurin to prevent relapse following allogeneic hematopoietic stem cell transplant (alloHSCT) in patients with acute myeloid leukemia (AML) harboring internal tandem duplication (ITD) in *FLT3*. Adults (aged 18–70 years) who received alloHSCT in first complete remission, had achieved hematologic recovery, and were transfusion independent were randomized to receive SOC with or without midostaurin (50 mg twice daily) continuously in twelve 4-week cycles. The primary endpoint was relapse-free survival (RFS) 18 months post-alloHSCT. Sixty patients were randomized (30/arm); 30 completed all 12 cycles (midostaurin + SOC, *n* = 16; SOC, *n* = 14). The estimated 18-month RFS (95% CI) was 89% (69–96%) in the midostaurin arm and 76% (54–88%) in the SOC arm (hazard ratio, 0.46 [95% CI, 0.12–1.86]; *P* = 0.27); estimated relapse rates were 11% and 24%, respectively. Inhibition of FLT3 phosphorylation to <70% of baseline (achieved by 50% of midostaurin-treated patients) was associated with improved RFS. The most common serious adverse events were diarrhea, nausea, and vomiting. Rates of graft-vs-host disease were similar between both arms (midostaurin + SOC, 70%; SOC, 73%). The addition of midostaurin maintenance therapy following alloHSCT may provide clinical benefit in some patients with *FLT3*-ITD AML. (ClinicalTrials.gov identifier: NCT01883362).

## Introduction

Acute myeloid leukemia (AML), the most common acute leukemia, is difficult to treat and has a poor prognosis, with a 5-year survival of ~25% [1, 2]. Multiple factors, including age, performance status (e.g., Eastern Cooperative Oncology Group), and cytogenetic and molecular features, affect treatment decisions and outcomes [3, 4]. Mutations in fms-like tyrosine kinase 3 (*FLT3*) are among the most common in AML and confer a poor prognosis with poor overall survival (OS) [5–7]. Consequently, these patients, particularly those with internal tandem duplications (ITDs), historically have more frequent and earlier relapses than patients without *FLT3* mutations [7, 8].

Midostaurin, a multikinase inhibitor that targets FLT3 and other kinases, was approved for the treatment of adult patients with newly diagnosed, *FLT3*-mutated AML when combined with intensive induction and consolidation chemotherapy [9]. Approval was based on the phase 3 RATIFY/CALGB 10603 trial, which demonstrated improved survival with the addition of midostaurin to intensive chemotherapy followed by single-agent maintenance therapy in patients aged <60 years with newly diagnosed, *FLT3*-mutated AML. The RATIFY trial did not allow patients receiving alloHSCT to continue midostaurin [10].

AlloHSCT in first complete remission (CR1) provides patients with *FLT3*-ITD-positive AML the highest likelihood of sustained remission [11, 12], but relapse rates remain high [13–15]. The prognosis for patients with *FLT3*-ITD mutations has been poor following standard alloHSCT, primarily because these patients have a higher risk of relapse than patients with *FLT3*-ITD-negative AML [14–16].

Post-HSCT maintenance therapy with tyrosine kinase inhibitors (TKIs) may improve outcomes in patients with *FLT3*-mutated AML. In a phase 2 trial (AMLSG 16-10), midostaurin combined with intensive chemotherapy followed by alloHSCT and single-agent maintenance therapy demonstrated improved rates of event-free survival in patients receiving midostaurin compared with historical controls [17]. In AMLSG 16-10, midostaurin was administered as in RATIFY; however, patients who underwent alloHSCT could resume midostaurin as maintenance therapy post-transplant [10, 17]. Data from phase 1 and 2 trials suggest there may be a benefit with sorafenib, another TKI, as maintenance therapy post-HSCT [18–20]. Results from the phase 2 SORMAIN trial, which evaluated post-alloHSCT maintenance with sorafenib, suggested a benefit with sorafenib versus placebo with a median 2-year relapse-free survival (RFS) rate of 85% (95% CI, 70–93%) vs 53% (95% CI, 37–68%), respectively, (hazard ratio [HR], 0.39 [95% CI, 0.183—0.848]; *P* = 0.013) [20]. Similarly, quizartinib, a FLT3 TKI, was safely administered after alloHSCT in a phase 1 study [21]. Detailed trials evaluating FLT3 TKIs as maintenance therapy are ongoing [22–25].

Here, we report the results of the RADIUS trial investigating whether the addition of midostaurin to standard-of-care (SOC) treatment post-alloHSCT improves RFS over SOC alone in patients with *FLT3*-ITD-positive AML.

## Patients and methods

### Study design

RADIUS (NCT01883362) was a phase 2, randomized, open-label trial of SOC with or without midostaurin in patients (aged 18–70 years) with documented *FLT3*-ITD-positive AML who had undergone a protocol-specified conditioning regimen before alloHSCT in CR1 (following hematologic recovery, transfusion independence, and controlled graft-vs-host disease [GVHD]). Patients were enrolled after engraftment and randomized 1:1 within 28 to 60 days after alloHSCT to receive SOC ± midostaurin (50 mg twice daily in twelve 4-week cycles). SOC was dictated by the treating physician but excluded alternate TKI therapy. Currently, SOC therapy varies per treating institution in the post-alloHSCT setting. SOC therapy includes anti-infective prophylaxis and treatment as well as GVHD prophylaxis and treatment along with supportive care. Anti-infective and GVHD prophylaxis treatments were based on institutional guidelines.

Patients were assessed for relapse and survival through 24 months post-alloHSCT and/or until the end of the study. Patient visits occurred monthly for 1 year during treatment and every other month during the 24-month follow-up. Adverse events (AEs) were tracked for 30 days after treatment ended and assessed per the Common Terminology Criteria for Adverse Events version 4.0 [26].

The study was performed in accordance with the International Council for Harmonisation Good Clinical Practice guidelines and the principles of the Declaration of Helsinki and was approved by institutional review boards at participating institutions. All patients provided written informed consent.

### Endpoints

The primary endpoint was RFS (time from transplant to relapse or death due to disease) 18 months after alloHSCT. Key secondary endpoints were safety, OS (time from transplant to the date of death from any cause), and RFS 24 months after alloHSCT.

Pharmacokinetics and in vivo FLT3 inhibition by FLT3 plasma inhibitory activity (PIA) assay were assessed as preplanned exploratory endpoints (see Supplementary methods). FLT3 inhibition and FLT3 ligand levels were evaluated on the basis of phosphorylated FLT3 (P-FLT3) and FLT3 ligand levels in the plasma [27].

The incidence and severity of GVHD were also exploratory study objectives. The percentage of patients developing acute or chronic GVHD (categorized according to the National Institutes of Health Consensus Development Project Working Group criteria [28]) and grade of GVHD were collected throughout the study by local assessment. GVHD by category and organ class was assessed at each study visit.

### Statistical analysis

RADIUS was an exploratory, signal-finding study not powered to detect a statistical difference between treatment arms. A sample size of 60 was calculated to detect a 50% reduction in the risk of relapse with 71% power, assuming a 15% incidence of relapse in the midostaurin arm.

For time-to-event analyses, Kaplan–Meier curves were used to estimate survival distributions. A Cox proportional hazards model was used to estimate the HR and associated 95% CIs.

## Results

### Patients

**Fig. 1:**
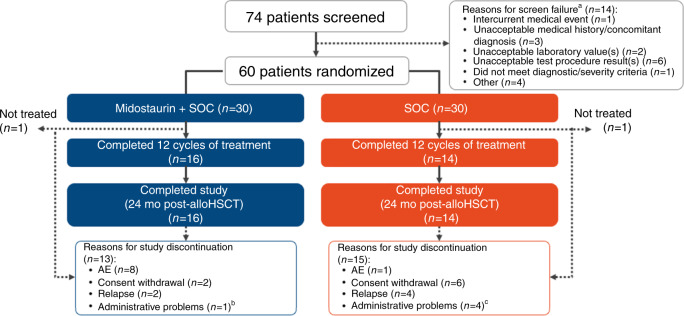
CONSORT diagram. AE adverse event, alloHSCT allogeneic hematopoietic stem cell transplant, SOC, standard of care. ^a^A single patient might have had >1 reason for screen failure. ^b^Early termination due to work schedule conflicts. ^c^Patients lost to follow-up (*n* = 2), early termination due to hospitalization at outside facility (*n* = 1), and early termination due to large travel distance (*n* = 1).

Between February 5, 2014, and June 13, 2016, 74 patients were screened and 60 patients (30 per arm) were randomized at 18 sites in the United States and 1 site in Canada (Fig. 1 and Table S1). All patients were in CR1 prior to transplant; 18 patients (30%) received transplant directly following induction, 39 (65%) of patients had received consolidation without additional maintenance, and 3 (5%) of patients had received pretransplant maintenance. All patients had completed a protocol-specified conditioning regimen before alloHSCT (Table S2). Overall, 30 patients completed the per-protocol 12 cycles of therapy (midostaurin + SOC: 16 patients [53%]; SOC: 14 patients [47%]). The number of patients discontinuing early from the study was comparable between arms (midostaurin + SOC, *n* = 13; SOC, *n* = 15); however, the reasons for treatment discontinuation differed by arm, with AEs being the most common reason in the midostaurin arm (27% vs 3%) and consent withdrawal being the most common reason in the SOC arm (7% vs 20%). Patients who withdrew from treatment were to return for relapse and follow-up assessments and were not considered withdrawn from the study. Patients who withdrew consent were censored at the time of withdrawal. Patient demographics, baseline characteristics, and transplant characteristics are shown in Table 1. Most patients (midostaurin + SOC, 100%; SOC, 90%) had de novo AML. The 2 arms were balanced with regard to age, sex, and race.

**Table 1:** Baseline patient and transplant characteristics.

Full analysis set	Midostaurin + SOC (*n* = 30)	SOC (*n* = 30)
Median age (range), years^a^	48 (20–61)	56 (20–68)
Sex, *n* (%)^b^		
Male	16 (53)	18 (60)
Female	14 (47)	12 (40)
Race, *n* (%)^c^		
White	27 (90)	27 (90)
Other	3 (10)	3 (10)
AML status at initial diagnosis, *n* (%)		
De novo	27 (90)	30 (100)
Secondary to AHD	1 (3)	0
Therapy related	2 (7)	0
Median peripheral WBC count (range), × 10^9^/L	48 (<1–278)	55 (<1–344)
Presence of *FLT3*-TKD mutation		
Yes	3 (10)	2 (7)
No	17 (57)	20 (67)
Unknown	10 (33)	8 (27)
Purpose of pre-HSCT treatment, *n* (%)		
Induction	30 (100)	30 (100)
Consolidation	22 (73)	20 (67)
Maintenance	2 (7)	1 (3)
Median time to randomization (range), days	54 (34–61)	54 (30–64)
Donor type, *n* (%)		
Syngeneic	0	1 (3)
Allogeneic, matched related^d^	10 (33)	14 (47)
Allogeneic, matched unrelated^d^	20 (67)	15 (50)
Stem cell source, *n* (%)		
Peripheral blood	29 (97)	28 (93)
Bone marrow	1 (3)	2 (7)

*AHD* antecedent hematologic disorder, *AML* acute myeloid leukemia, *FLT3* fms-like tyrosine kinase 3, *HLA* human leukocyte antigen, *HSCT* hematopoietic stem cell transplant, *SOC* standard of care, *TKD* tyrosine kinase domain, *WBC* white blood cell.

^a^*P* = 0.14; 2-sample *t*-test.

^b^*P* = 0.60; Fisher exact test.

^c^*P* = 0.72; Fisher exact test.

^d^Matched donors had HLA typing to include an 8/8 or 7/8 allele match rate at HLA-A, -B, -C, and -DRB1. A single mismatch was allowed.

### Efficacy

**Fig. 2:**
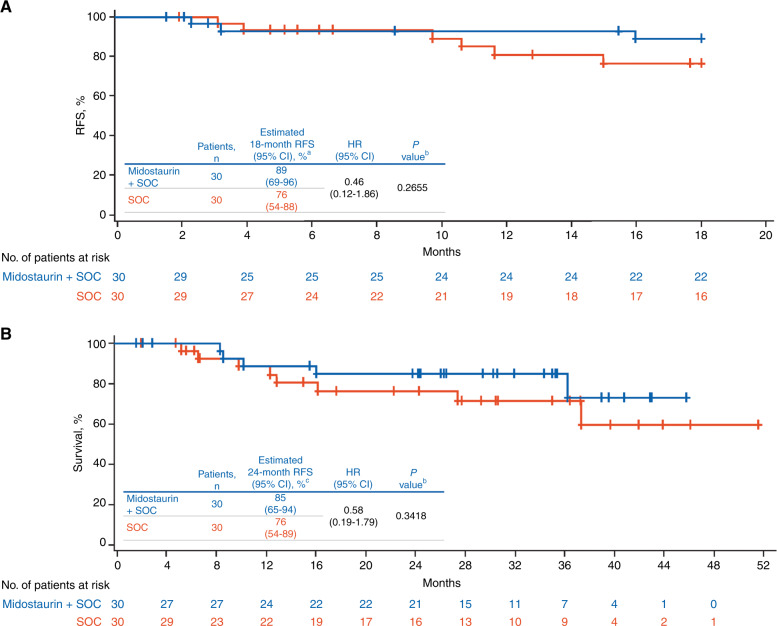
Outcomes after alloHSCT. Kaplan–Meier curves of **A** RFS by treatment arm at 18 months after undergoing alloHSCT and **B** OS by treatment arm at 24 months after undergoing alloHSCT. Blue, midostaurin + SOC; red, SOC. Tick marks indicate censoring of data. alloHSCT allogeneic hematopoietic stem cell transplant, HR hazard ratio, OS overall survival, RFS relapse-free survival, SOC standard of care. ^a^Median RFS was not reached. ^b^Log-rank *P* value. ^c^Median OS was not reached.

The estimated RFS at 18 months (95% CI) was 89% (69–96%) with midostaurin and 76% (54–88%) with SOC alone (HR, 0.46 [95% CI, 0.12–1.86]; *P* = 0.27) (Fig. 2A). There were 3 RFS events in the midostaurin arm and 6 RFS events in the SOC arm at 18 months. The predicted relative reduction in the risk of relapse with the addition of midostaurin was 54% at 18 months post-alloHSCT.

At 24 months, addition of midostaurin to SOC continued to demonstrate reduced risk of relapse and prolonged survival compared with SOC alone (Figs. 2B and S1). At the time of final analysis (i.e., when all patients who remained on the study had reached 24 months post-alloHSCT), the median RFS and OS were not reached in either treatment arm. There were 4 relapses (13%) in the midostaurin arm vs 5 relapses (17%) in the SOC arm; median time to relapse from transplant was similar across both arms (median [range]; midostaurin + SOC, 323.5 days [69–1028 days]; SOC alone, 323 days [94–456 days]). The estimated 24-month RFS (95% CI) was 85% (64–94%) with midostaurin and 76% (54–88%) with SOC alone (HR, 0.60 [95% CI, 0.17–2.14]; *P* = 0.4297), and the relative reduction in the risk of relapse with the addition of midostaurin remained high at 40%.

Survival outcomes also improved; the estimated 24-month OS (95% CI) was 85% (65%-94%) with midostaurin and 76% (54%-89%) with SOC alone (HR, 0.58 [95% CI, 0.19–1.79]; *P* = 0.34), which is a 42% reduction in the risk of death with the addition of midostaurin (albeit not statistically significant). Eight patients died in the SOC arm vs 5 patients in the midostaurin arm; relapse accounted for a similar fraction of deaths in each arm. Details of post-relapse treatment were not captured. A total of 7 patients died due to reasons other than relapse: 5 in the SOC arm and 2 in the midostaurin arm; these patients were censored at the date of death. Non-relapse mortality was due to study indication (*n* = 2) and 1 instance each of cardiac arrest, GVHD, hepatic failure, cardiopulmonary arrest, and encephalitis infection.

### Pharmacokinetics and PIA assay

The pharmacokinetics of midostaurin and its main metabolites (CGP62221 and CGP52421) were evaluated in 29 patients. The mean plasma concentration of midostaurin reached a maximum duringcycle 1 day 15, where as CGP52421 and CGP62221 peaked at cycle 3 day 1; all reached steady-state levels at cycle 4 (Fig. S2).

**Fig. 3:**
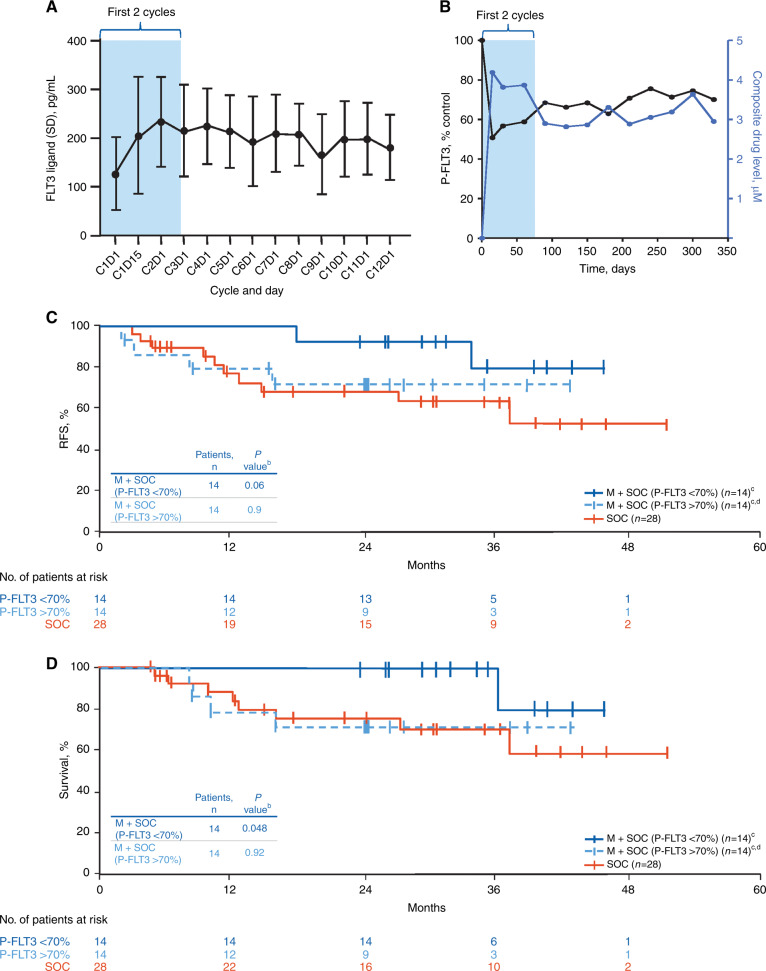
Correlation between exploratory biomarker analyses and clinical outcomes. **A** Median FLT3 ligand levels and **B** median P-FLT3 levels relative to baseline and concurrent combined levels of midostaurin and its metabolites in patients who received midostaurin + SOC. Median P-FLT3 levels were 70% of baseline at C3D1. **C** RFS and **D** OS at 24 months after alloHSCT in patients who received midostaurin + SOC stratified by P-FLT3 level (<70% vs >70%). **C** cycle; **D** day; FLT3, fms-like tyrosine kinase 3; M midostaurin, P-FLT3 phosphorylated FLT3, OS overall survival, RFS relapse-free survival, SOC standard of care. ^a^For this analysis, RFS was defined as time from transplant to relapse or death from any cause. ^b^Log-rank *P* value vs SOC (*n* = 28). ^c^Patients who reached C3D1 and received midostaurin + SOC (*n* = 28) were stratified according to FLT3 inhibition levels above or below the median (median P-FLT3, 70%). FLT3 inhibition was higher in patients with P-FLT3 levels <70% of baseline. ^d^P-FLT3 > 70% includes patients with missing P-FLT3 at C3D1.

Among patients who received midostaurin, 28 were evaluable using the PIA assay. The degree of P-FLT3 inhibition was greatest during the first 2 cycles of therapy (Fig. 3A, B). In an exploratory biomarker analysis that assessed the correlation between plasma levels of midostaurin and its primary metabolites with the degree of FLT3 inhibition (i.e., lower levels of P-FLT3), early inhibition of FLT3 correlated inversely with drug levels (Fig. 3B). Peak FLT3 inhibition occurred at cycle 3 day 1; this time point was chosen for the correlative analysis.

In patients receiving midostaurin (*n* = 28), the median P-FLT3 level at cycle 3 day 1 was 70% of baseline P-FLT3 levels. Thus, 14 of these patients had more effective inhibition of FLT3 activity (i.e., P-FLT3 levels <70% of baseline) on cycle 3 day 1 with P-FLT3 levels ranging from 20% to 69%. Of these 14 patients, 10 completed all 12 cycles of midostaurin therapy (Fig. S3). Among the remaining 14 patients who had less effective inhibition of FLT3 activity (i.e., P-FLT3 levels >70% of baseline), P-FLT3 was not measured at cycle 3 day 1 in 8 patients (6 were not receiving midostaurin on cycle 3 day 1). Six of 14 patients completed 12 cycles of midostaurin therapy and had P-FLT3 levels ranging from 74% to 100%. These higher P-FLT3 levels indicate less effective FLT3 inhibition, possibly resulting from the biological response of the patient to midostaurin or likely related to patient adherence to midostaurin, indicating the importance of proactive AE management to support patients throughout treatment.

Stratifying patients who received midostaurin by levels of FLT3 inhibition above or below the median revealed an association with clinical outcomes. Higher levels of FLT3 inhibition correlated with prolonged RFS, a reduced risk of relapse (*P* = 0.06), and significantly improved survival (*P* = 0.048) (Fig. 3C, D). Patients with less FLT3 inhibition had a similar risk of relapse and survival rate to those observed in patients receiving SOC alone (*P* = 0.9 and *P* = 0.92, respectively).

### Safety

With midostaurin + SOC and SOC alone, AEs occurred in 100% and 87% of patients, respectively (Table 2). Most AEs in both arms were grade 1/2. The most common AEs were low-grade gastrointestinal AEs (grades 1–3, midostaurin arm vs SOC arm): vomiting (73% vs 23%), nausea (67% vs 27%), and diarrhea (49% vs 23%). Gastrointestinal AEs were more common in the midostaurin arm than in the SOC arm. The most common grade 3/4 laboratory abnormalities, increased alanine aminotransferase, increased aspartate aminotransferase, and decreased neutrophils, occurred in both arms. Serious AEs (Table 3) occurred in 57% of patients with midostaurin and 30% of patients with SOC alone. The most common serious AEs (midostaurin arm vs SOC arm) were diarrhea (13% vs 7%), nausea and vomiting (both, 3% vs 10%), and pyrexia (7% vs 7%).

**Table 2:** Most common AEs (occurring in ≥15% of patients).

AE, *n* (%)	Midostaurin + SOC (*n* = 30)		SOC (*n* = 30)	
	Any grade	Grade ≥ 3	Any grade	Grade ≥ 3
Vomiting	7 (23)	1 (3)	22 (73)	2 (7)
Nausea	8 (27)	3 (10)	20 (67)	1 (3)
Diarrhea	7 (23)	1 (3)	12 (40)	3 (10)
Fatigue	9 (30)	0	8 (27)	1 (3)
Peripheral edema	9 (30)	0	8 (27)	0
Headache	7 (23)	0	8 (27)	0
Cough	6 (20)	0	8 (27)	0
ALT increased	7 (23)	4 (13)	6 (20)	3 (10)
Anemia	6 (20)	2 (7)	7 (23)	3 (10)
AST increased	8 (27)	4 (13)	5 (17)	2 (7)
Pruritus	6 (20)	0	7 (23)	3 (10)
Dry eye	6 (20)	0	5 (17)	0
Pyrexia	5 (17)	1 (3)	4 (20)	0
Rash	6 (20)	0	6 (17)	0
Tremor	4 (13)	0	7 (23)	0
Dyspnea	7 (23)	1 (3)	3 (10)	0
Insomnia	6 (20)	0	4 (13)	0
Neutrophil count decreased	3 (10)	2 (7)	7 (23)	4 (13)
Arthralgia	6 (20)	1 (3)	3 (10)	0
Dizziness	6 (20)	0	3 (10)	0
Hypertension	6 (20)	4 (13)	3 (10)	0
Upper respiratory tract infection	6 (20)	0	3 (10)	0

*AE* adverse event, *ALT* alanine aminotransferase, *AST* aspartate aminotransferase, *SOC* standard of care.

**Table 3:** Serious AEs occurring in ≥1 of patients overall.

AE, *n* (%)	Midostaurin + SOC (*n* = 30)	SOC (*n* = 30)
Diarrhea	4 (13)	2 (7)
Nausea	1 (3)	3 (10)
Vomiting	1 (3)	3 (10)
Pyrexia	2 (7)	2 (7)
Deep vein thrombosis	1 (3)	2 (7)
Febrile neutropenia	1 (3)	2 (7)
Anemia	2 (7)	1 (3)
Acute kidney injury	0	2 (7)
Abdominal pain	1 (3)	1 (3)
Parainfluenza virus infection	1 (3)	1 (3)

*AE* adverse event, *SOC* standard of care.

Median midostaurin exposure was 10.5 months (range, 0.2–11.5 months; defined by time of last midostaurin dose); 16 patients completed all 12 cycles of treatment. The median dose intensity was 93 mg/day (range, 25–100 mg/day). Dose adjustments were required per protocol in 19 patients (63%), most commonly due to AEs (84%). AEs leading to dose adjustment in ≥10% of patients included vomiting (27%), nausea (20%), and aspartate aminotransferase levels increased (10%). One patient was reported to have received a modified dose of midostaurin due to concomitant posaconazole, a cytochrome P450 3A4 inhibitor, per protocol.

AEs resulted in discontinuation from the study in 9 patients: 8 (27%) in the midostaurin arm and 1 (3%) in the SOC arm. The 8 patients in the midostaurin arm who discontinued treatment had 9 events: nausea (*n* = 3), vomiting (*n* = 2), liver function test levels increased (*n* = 2), pulmonary mycosis (*n* = 1), and pneumonitis (*n* = 1). The patient in the SOC arm discontinued from the study due to hypoxia. Twelve patients died on study during the follow-up phase (midostaurin + SOC, *n* = 4; SOC, *n* = 8). Death due to AML disease progression occurred in 2 patients receiving midostaurin and 4 receiving SOC alone. The addition of midostaurin to SOC did not result in an increase in the severity or rate of acute or chronic GVHD (Table 4). Rates of GVHD, as determined by local assessment, were similar between the midostaurin and SOC arms (overall, 70% vs 73%; acute, 53% vs 50%; and chronic, 37% vs 33%, respectively). Ninety-seven percent of patients received concomitant medication for the management of GVHD, including 28 (93%) in the midostaurin arm and 30 (100%) in the SOC arm. The most common concomitant medications typical of GVHD management were calcineurin inhibitors (85%), glucocorticoids (57%), moderately potent corticosteroids (18%), and selective immunosuppressants (17%) (Table S3).

**Table 4:** Incidence of GVHD.

GVHD, *n* (%)^a^	Midostaurin + SOC (*n* = 30)	SOC (*n* = 30)
Acute	15 (50)	16 (53)
Grade I	7 (23)	4 (13)
Grade II	8 (27)	10 (33)
Grade III	0	2 (7)
Grade IV	0	0
Chronic	9 (30)	10 (33)
Mild	2 (7)	5 (17)
Moderate	5 (17)	4 (13)
Severe	2 (7)	1 (3)

*GVHD* graft-vs-host disease, *SOC* standard of care.

^a^Patients could be counted in multiple categories.

The most common organ toxicity due to GVHD was localized to the skin and affected 50% of patients in the midostaurin arm and 47% of patients in the SOC arm (Fig. S4). All patients with skin involvement in the midostaurin arm had stage 1 or 2 disease, whereas 2 patients in the SOC arm experienced stage 3 disease. Neither arm reported stage 4 organ involvement. Upper gastrointestinal toxicity was similar in both groups and did not exceed stage 1. Lower gastrointestinal toxicity was reported only in patients in the SOC arm and was primarily stage 1.

## Discussion

This is the first randomized study of midostaurin as maintenance therapy after alloHSCT. We show that for patients with *FLT3*-ITD-positive AML in CR1, a defined course of up to 12 months of maintenance therapy with midostaurin was safely added to SOC after recovery from alloHSCT and improved RFS at 18 months after alloHSCT by 13% (over SOC alone). Although the study was not powered to detect a treatment difference, there was a trend toward benefit with midostaurin for all efficacy endpoints evaluated.

The survival outcomes in all participants in this study were better than anticipated for this high-risk leukemia population. Historically, the expected 2-year OS with SOC was closer to 60% compared with 76% observed in this study [15]. The stringent enrollment criteria, including recovery of counts (i.e., absolute neutrophil count >1000/μL and platelet count ≥20,000/μL without platelet transfusion) by day 42, ability to start treatment by day 60 post- alloHSCT, and no active, advanced, acute GVHD, may have contributed to the survival outcomes observed for all participants in this study. Moreover, the median time from the date of alloHSCT to initiation of study drug for both arms was 54 days; patients who had morphological relapse before that date were ineligible. Consistently, factors related to these inclusion/exclusion criteria, such as unacceptable test procedure results (8%) and unacceptable medical history/concomitant diagnosis (4%), were common reasons for screen failure, though the overall rate of screen failures (14 of 74 patients screened [19%]) was relatively low. Censoring of patients at the date of death due to non-relapse mortality may also have contributed to survival rates, particularly given the small patient population in this study. Similarly, patients were not stratified by European LeukemiaNet or National Comprehensive Cancer Network molecular risk classification due to the size of the study; thus, enrollment of patients with favorable molecular risk factors may also have affected the survival rates observed.

Correlative analysis suggests that patients who tolerated midostaurin and remained on therapy, as demonstrated by relatively higher levels of P-FLT3 inhibition, may have sustained benefit and long-term outcomes. The PIA assay allows for an indirect measurement of the phosphorylation of FLT3. P-FLT3 inhibition to <70% of baseline was achieved by 50% of patients receiving midostaurin and was associated with improved RFS and OS, indicating that inhibiting FLT3, even modestly, can have clinical benefit. Treatment adherence was not uniform in all patients receiving midostaurin, possibly due to tolerability (e.g., gastrointestinal toxicity). Prophylactic support, including antiemetics, in the management of gastrointestinal toxicities was crucial in keeping patients on therapy to provide the clinical benefit suggested by these data. Thus, increases in gastrointestinal toxicities were primarily low grade and manageable, consistent with other reports with single-agent midostaurin [29, 30]. Addition of midostaurin to SOC did not increase rates or severity of GVHD. Although the PIA assay is not used in clinical practice, FLT3 inhibition measured by this assay has tightly correlated with clinical activity across a broad array of FLT3 inhibitors [27, 31–34]. The results from the exploratory analysis in this study suggest that midostaurin therapy after alloHSCT may provide high levels of FLT3 inhibition in the long term in patients who remain on treatment, though further validation is required.

These data are consistent with the safety profile of midostaurin in patients with *FLT3*-ITD AML. In line with the AMLSG 16-10 study [17], the median time of midostaurin exposure during maintenance was similar (9 months in AMLSG 16-10 and 10 months in RADIUS); discontinuation due to toxicity was more common in AMLSG 16-10 (55%) than in RADIUS (27%), which may be explained by the stringent inclusion criteria of RADIUS. However, both studies demonstrated the safety and feasibility of midostaurin maintenance therapy.

Post-alloHSCT maintenance therapy with FLT3 TKIs, including midostaurin, is a viable treatment for reducing the risk of relapse in patients with *FLT3*-ITD AML. We anticipate that this study will provide a landmark for future studies, as the population had no pretransplant TKI exposure. These results complement those of the AMLSG 16-10 trial, which demonstrated improved event-free survival for patients with *FLT3*-ITD AML who received pretransplant midostaurin and began midostaurin within 100 days post-transplant compared with patients who only received pretransplant midostaurin [17]. Evidence from the present study and AMLSG 16-10 suggest that midostaurin maintenance therapy may be most appropriate for patients aged 18–70 years with *FLT3*-ITD AML who have undergone alloHSCT in CR1 and can begin midostaurin therapy quickly (within 100 days, ideally <60 days).

With the approval of midostaurin as up-front therapy for *FLT3*-ITD AML, new trials are emerging to better clarify the role of post-transplant TKI therapy in patients with deeper molecular remission, such as the large, phase 3, multinational, randomized trial assessing gilteritinib vs placebo as post-transplant adjuvant therapy for patients with *FLT3*-ITD AML in CR1 (BMT-CTN 1506; NCT02997202). As available treatment options increase, more detailed scrutiny of the risk-benefit profiles of these targeted agents is likely to be required.

With a post-transplant 2-year OS of ~80%, this study highlights the impact of recent advances in the management of *FLT3*-ITD AML on survival outcomes. Because *FLT3-*mutated AML has a higher risk of relapse than *FLT3*-mutation-negative AML, the addition of midostaurin maintenance therapy post-HSCT may be a viable option to reduce the risk of relapse in some patients after alloHSCT. These results provide evidence of clinical benefit and an estimate of treatment effect that could inform larger-scale studies in the future.

## Supplementary information

Supplemental Material
