# Supplementary material for: Midostaurin after allogeneic stem cell transplant in patients with FLT3-internal tandem duplication-positive acute myeloid leukemia
Source: Bone Marrow Transplant. 2020 Dec 7;56(5):1180–9. doi: 10.1038/s41409-020-01153-1 (PMC8113057; doi:10.1038/s41409-020-01153-1)
Supplement: Supplementary file 1 — Supplemental Material [file 41409_2020_1153_MOESM1_ESM.docx]

**Midostaurin after allogeneic stem cell transplant in patients with *FLT3*-internal tandem duplication–positive acute myeloid leukemia**

Richard T. Maziarz, Mark Levis, Mrinal M. Patnaik, Bart L. Scott, Sanjay R. Mohan, Abhinav Deol, Scott D. Rowley, Dennis D.H. Kim, Daniela Hernandez, Trivikram Rajkhowa, Kelly Haines, Gaetano Bonifacio, Patrice Rine, Das Purkayastha, and Hugo F. Fernandez

# Supplemental Methods

*Study Design and Powering*

The planned sample size of 30 patients per treatment arm was chosen to enable separate estimates of the primary relapse-free survival (RFS) endpoint. Given the rarity of patients with fms-like tyrosine kinase 3 internal tandem duplication–positive (FLT3-ITD+) acute myeloid leukemia who have received an allogeneic hematopoietic stem cell transplant (alloHSCT) and are in first complete remission (CR1) (FLT3-ITD mutation rate: approximately 22-25%; alloHSCT rate: approximately 25%; as estimated in the phase 3 RATIFY trial),^1^ powering of this study was not feasible.

A total of 74 patients were screened, and there were 14 (19%) screen failures. Common reasons for screen failure included unacceptable test procedure results (8%) and unacceptable medical history/concomitant diagnosis (4%). The full list of reasons for screen failure is shown in Figure 1.

*Sample Collection*

| **Sample Collection Frequency** | |
| --- | --- |
| **Sample Type** | **Collection Time(s)** |
| Hematology lab panels | Every 2 months (to assess relapse and survival) |
| Bone marrow aspirations | Early (30-100 days post-alloHSCT), at cycles 6 and 12, and at relapse (or more frequently if clinically indicated) |
| Blood samples for plasma inhibitory activity assay | Day 1 of every cycle (≤12) and day 15 of cycle 1 |

*Plasma Inhibitory Activity Assay*

Pharmacokinetics and in vivo FLT3 inhibition by FLT3 plasma inhibitory activity (PIA) assay were assessed as preplanned exploratory endpoints. The degree of in vivo FLT3 inhibitor as assessed by the PIA assay has been correlated with clinical response for patients receiving FLT3 inhibitors.^2, 3^ FLT3 inhibition and FLT3 ligand levels were evaluated on the basis of phosphorylated FLT3 (P-FLT3) and FLT3 ligand levels in the plasma.^4^ Blood samples collected at specified time points were fractionated and frozen. For the PIA assay, frozen plasma was thawed and incubated with the FLT3-ITD–expressing Molm14 cell line. Immunoblotting was performed for P-FLT3, and an enzyme-linked immunosorbent assay was used to evaluate FLT3 ligand levels in the plasma, as previously described.^4, 5^

*End of Treatment and Discontinuation*

Per protocol, patients may have withdrawn voluntarily or at investigator discretion at any time. Reasons for treatment withdrawal may have included adverse events, abnormal laboratory values or test procedure results, withdrawn consent, loss to follow-up, administrative problems, death or relapse, completion of per-protocol duration of treatment, or deviation from protocol. Patients who withdrew from treatment were to return for relapse and follow-up assessments and were not considered withdrawn from the study. Patients with non-relapse mortality were censored at the date of death.

In the standard of care arm, end of treatment was defined as the end of cycle 12 or date of first relapse/discontinuation. In the midostaurin arm, end of treatment was defined as the date of last midostaurin dose.

# Supplemental Results

## Disease-Free Survival

Disease-free survival (DFS), a key secondary endpoint, was defined as the time from transplant to relapse or death from any cause. At the time of final analysis (ie, when all patients who remained on the study had reached 24 months post-allogeneic hematopoietic stem cell transplant), the median DFS was not reached for either treatment arm. There was a 45% relative reduction in the risk of disease relapse with the addition of midostaurin, with the estimated DFS at 24 months (95% CI) of 81% (61%-92%) with midostaurin and 69% (48%-83%) with standard of care alone (HR, 0.55 [95% CI, 0.20-1.52]; *P*=.24) (Figure S1B).

# Supplemental Tables

**Table S1.** Study sites and number of patients enrolled at each site.

| Study Site | No. of Patients Enrolled |
| --- | --- |
| Oregon Health & Sciences University  3181 SW Sam Jackson Park Rd  Portland, OR 97239 | 8 |
| Mayo Clinic  200 First St SW  Rochester, MN 55905 | 7 |
| Fred Hutchinson Cancer Research Center  1100 Fairview Avenue North  Seattle, WA 98122 | 5 |
| Barbara Ann Karmanos Cancer Institute  4100 John R  Detroit, MI 48201 | 5 |
| Moffitt Cancer Center  12902 Magnolia Dr  Tampa, FL 33612 | 4 |
| Princess Margaret Hospital  610 University Ave  Toronto, Ontario, Canada  M5G 2M9 | 4 |
| Henry-Joyce Cancer Clinic  1301 Medical Center Dr  3927 The Vanderbilt Clinic  Nashville, TN 37232 | 4 |
| Hackensack University Medical Center  30 Prospect Ave  Hackensack, NJ 07601 | 4 |
| Texas Oncology-Baylor Charles A. Sammons Cancer Center  3410 Worth St  Dallas, TX 75246 | 3 |
| Blood and Marrow Transplant Group of Georgia  5670 Peachtree Dunwoody Rd, Suite 1000  Atlanta, GA 30342 | 3 |
| The Sarah Cannon Research Institute  250 25th Ave N  Suite 412, Administration  Nashville, TN 37203 | 2 |
| Washington University School of Medicine  Siteman Cancer Center  4921 Parkview Pl  7th Floor  St Louis, MO 63110 | 2 |
| The University of Chicago Medical Center  5841 S Maryland Ave  Chicago, IL 60637 | 2 |
| UC San Diego Moores Cancer Center  3855 Health Sciences Dr  MC 0960  La Jolla, CA 92093-0960 | 2 |
| Ronald Reagan UCLA Cancer Center  757 Westwood Blvd  Los Angeles, CA 90095 | 1 |
| Colorado Blood Cancer Institute  1721 E 19th Ave, Suite 300  Denver, CO 80218 | 1 |
| Methodist Healthcare System of San Antonio  Methodist Hospital  7700 Floyd Curl Dr  San Antonio, TX 78229 | 1 |
| UNC Health Care  NC Cancer Hospital  101 Manning Drive  Chapel Hill, NC 27599 | 1 |
| University Hospitals Cleveland  11100 Euclid Ave  Cleveland, OH 44106 | 1 |

**Table S2.** Summary of pre-alloHSCT conditioning regimen.

| Regimen, n (%) | Midostaurin + SOC (N=30) | SOC (N=30) | Total (N=60) |
| --- | --- | --- | --- |
| Busulfan/cyclophosphamide | 13 (43.3) | 8 (26.7) | 21 (35.0) |
| Busulfan/fludarabine | 14 (46.7) | 17 (56.7) | 31 (51.7) |
| Cyclophosphamide/total body irradiation | 1 (3.3) | 2 (6.7) | 3 (5.0) |
| Fludarabine/melphalan | 1 (3.3) | 3 (10.0) | 4 (6.7) |

SOC, standard of care.

**Table S3.** Concomitant medications administered for the management of GVHD.

| **ATC Class (ATC Level 4) Preferred Term, n (%)** | **Midostaurin + SOC (N=30)** | **SOC (N=30)** | **Total (N=60)** |
| --- | --- | --- | --- |
| Any concomitant medications taken for management of GVHD^a^ | 28 (93.3) | 30 (100) | 58 (96.7) |
| Aminoalkyl ethers  Diphenhydramine | 1 (3.3)  1 (3.3) | 0  0 | 1 (1.7)  1 (1.7) |
| Antibiotics  Nystatin | 1 (3.3)  1 (3.3) | 0  0 | 1 (1.7)  1 (1.7) |
| Antiinfectives and antiseptics for local oral treatment  Chlorhexidine | 0  0 | 1 (3.3)  1 (3.3) | 1 (1.7)  1 (1.7) |
| Bile acid preparations  Ursodeoxycholic acid | 4 (13.3)  4 (13.3) | 1 (3.3)  1 (3.3) | 5 (8.3)  5 (8.3) |
| Calcineurin inhibitors  Cyclosporine  Tacrolimus | 25 (83.3)  7 (23.3)  21 (70.0) | 26 (86.7)  6 (20.0)  20 (66.7) | 51 (85.0)  13 (21.7)  41 (68.3) |
| Caries prophylactic agents  Sodium fluoride | 1 (3.3)  1 (3.3) | 0  0 | 1 (1.7)  1 (1.7) |
| Corticosteroids acting locally  Beclometasone | 0  0 | 1 (3.3)  1 (3.3) | 1 (1.7)  1 (1.7) |
| Corticosteroids for local oral treatment  Dexamethasone | 2 (6.7)  2 (6.7) | 1 (3.3)  1 (3.3) | 3 (5.0)  3 (5.0) |
| Corticosteroids, moderately potent (Group II)  Dexamethasone  Triamcinolone  Triamcinolone acetonide | 5 (16.7)  1 (3.3)  0  4 (13.3) | 6 (20.0)  2 (6.7)  3 (10.0)  3 (10.0) | 11 (18.3)  3 (5.0)  3 (5.0)  7 (11.7) |
| Corticosteroids, plain  Prednisolone acetate | 0  0 | 1 (3.3)  1 (3.3) | 1 (1.7)  1 (1.7) |
| Corticosteroids, potent (Group III)  Fluocinonide | 0  0 | 1 (3.3)  1 (3.3) | 1 (1.7)  1 (1.7) |
| Corticosteroids, very potent (Group IV)  Clobetasol | 2 (6.7)  2 (6.7) | 2 (6.7)  2 (6.7) | 4 (6.7)  4 (6.7) |
| Corticosteroids, weak (Group I)  Hydrocortisone | 1 (3.3)  1 (3.3) | 2 (6.7)  2 (6.7) | 3 (5.0)  3 (5.0) |
| Fluoroquinolones  Levofloxacin | 0  0 | 2 (6.7)  2 (6.7) | 2 (3.3)  2 (3.3) |
| Glucocorticoids  Beclometasone  Budesonide  Dexamethasone  Fluticasone propionate  Methylprednisolone  Methylprednisolone sodium succinate  Prednisone | 16 (53.3)  3 (10.0)  4 (13.3)  2 (6.7)  0  2 (6.7)  2 (6.7)  11 (36.7) | 18 (60.0)  3 (10.0)  6 (20.0)  1 (3.3)  1 (3.3)  7 (23.3)  1 (3.3)  11 (36.7) | 34 (56.7)  6 (10.0)  10 (16.7)  3 (5.0)  1 (1.7)  9 (15.0)  3 (5.0)  22 (36.7) |
| Leukotriene receptor antagonists  Montelukast | 0  0 | 1 (3.3)  1 (3.3) | 1 (1.7)  1 (1.7) |
| Macrolides  Azithromycin | 0  0 | 1 (3.3)  1 (3.3) | 1 (1.7)  1 (1.7) |
| Monoclonal antibodies  Rituximab | 0  0 | 1 (3.3)  1 (3.3) | 1 (1.7)  1 (1.7) |
| Other antihistamines for systemic use  Hydroxyzine hydrochloride | 1 (3.3)  1 (3.3) | 0  0 | 1 (1.7)  1 (1.7) |
| Other immunosuppressants  Azathioprine | 1 (3.3)  1 (3.3) | 0  0 | 1 (1.7)  1 (1.7) |
| Other ophthalmologicals  Cyclosporine  No ingredients/substances | 1 (3.3)  0  1 (3.3) | 1 (3.3)  1 (3.3)  0 | 2 (3.3)  1 (1.7)  1 (1.7) |
| Other parasympathomimetics  Cevimeline hydrochloride | 0  0 | 1 (3.3)  1 (3.3) | 1 (1.7)  1 (1.7) |
| Proton pump inhibitors  Pantoprazole | 0  0 | 1 (3.3)  1 (3.3) | 1 (1.7)  1 (1.7) |
| Selective immunosuppressants  Mycophenolate mofetil  Sirolimus | 2 (6.7)  1 (3.3)  1 (3.3) | 8 (26.7)  5 (16.7)  3 (10.0) | 10 (16.7)  6 (10.0)  4 (6.7) |
| Synthetic anticholinergics, esters with tertiary amino group  Dicycloverine | 0  0 | 1 (3.3)  1 (3.3) | 1 (1.7)  1 (1.7) |
| Not coded  No ingredients/substances | 0  0 | 2 (6.7)  2 (6.7) | 2 (3.3)  2 (3.3) |

ATC, Anatomical Therapeutic Chemical; GVHD, graft-vs-host disease; SOC, standard of care.

^a^ A single patient may have received >1 concomitant medication for the management of GVHD over the course of treatment.

# Supplemental Figures

**Figure S1.** Kaplan-Meier curves of (A) relapse-free survival (RFS) and (B) disease-free survival (DFS) by treatment arm at 24 months after alloHSCT. Blue, midostaurin + SOC; red, SOC. Tick marks indicate censoring of data. alloHSCT, allogeneic hematopoietic stem cell transplant; HR, hazard ratio; SOC, standard of care. ^a^ Median RFS was not reached. ^b^ Log-rank *P* value. ^c^ Median DFS was not reached.

**A.** RFS: 24 months after alloHSCT


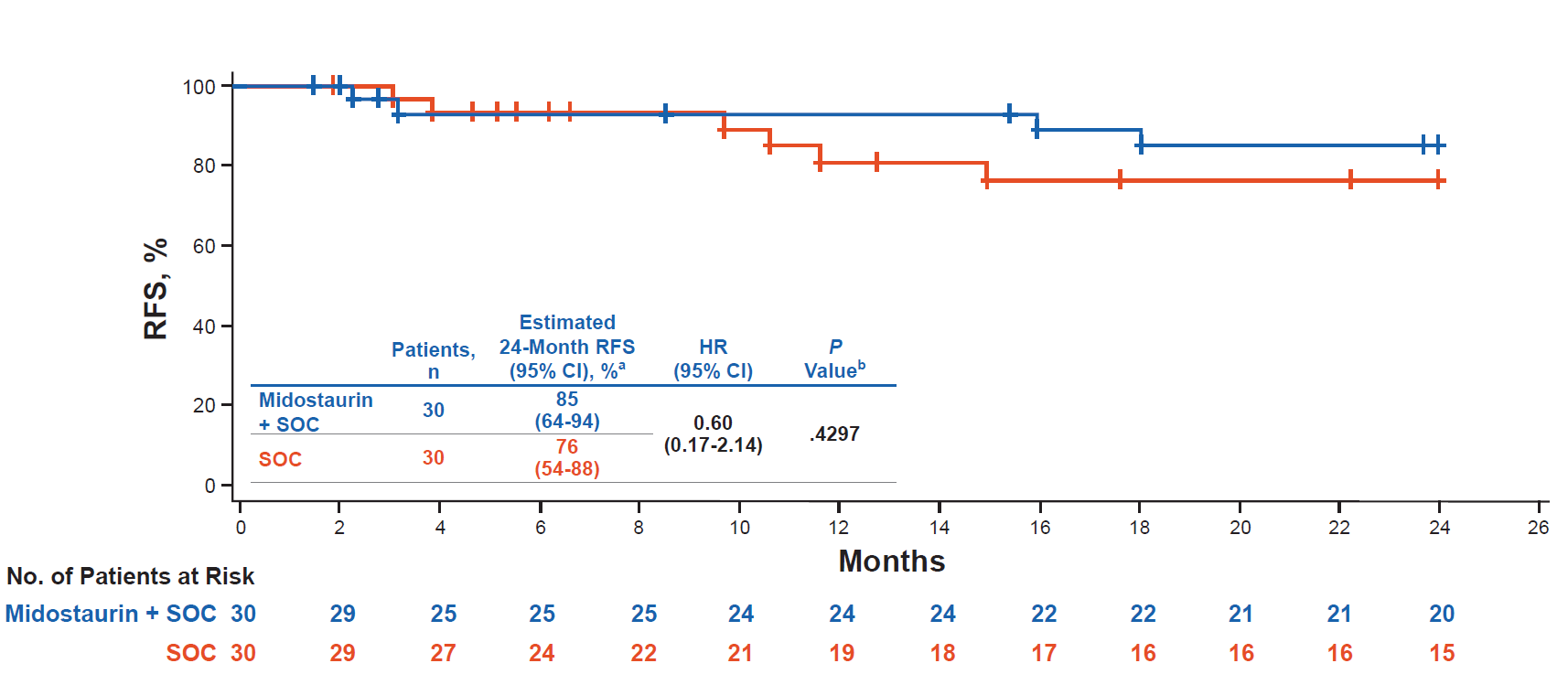


**B.** DFS 24 months after alloHSCT


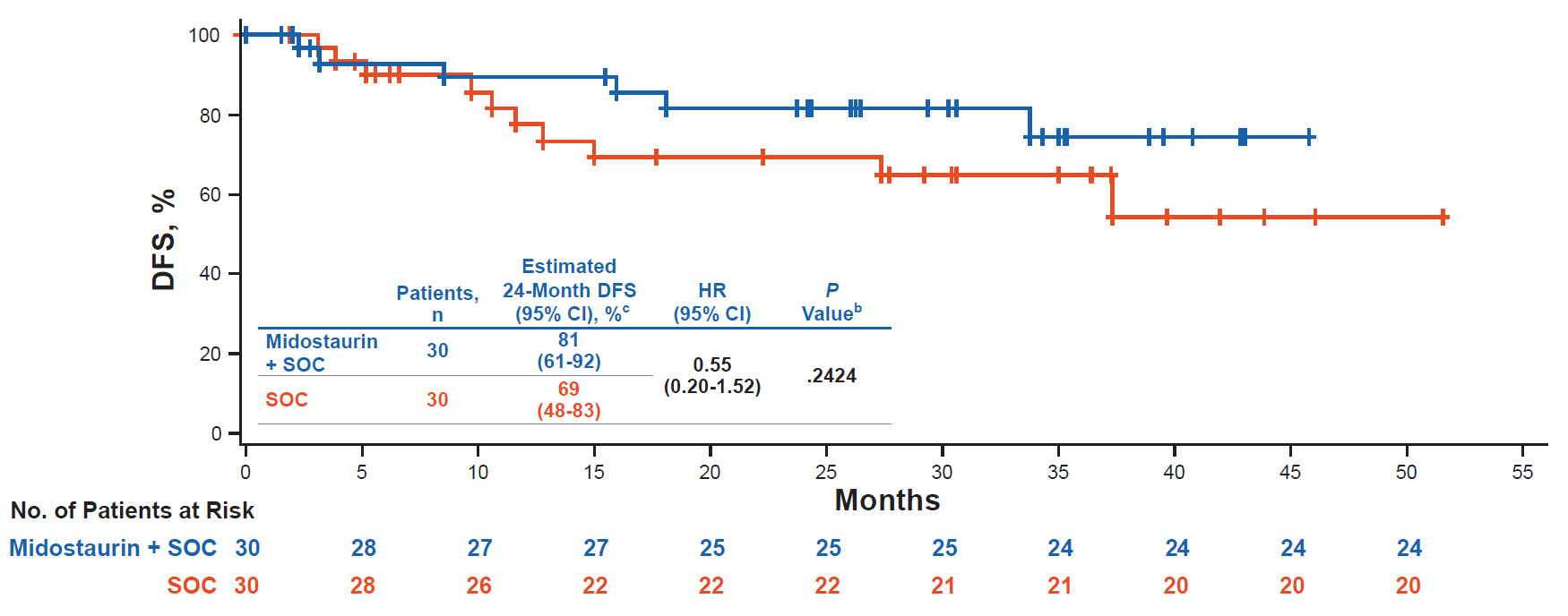


**Figure S2.** Pharmacokinetics of midostaurin and its metabolites (CGP52421 and CGP62221). Plasma concentration of (A) midostaurin, (B) CGP52421, and (C) CGP62221 in patients who received midostaurin + standard of care and for whom samples were available (n=29). C, cycle; D, day.

**A**


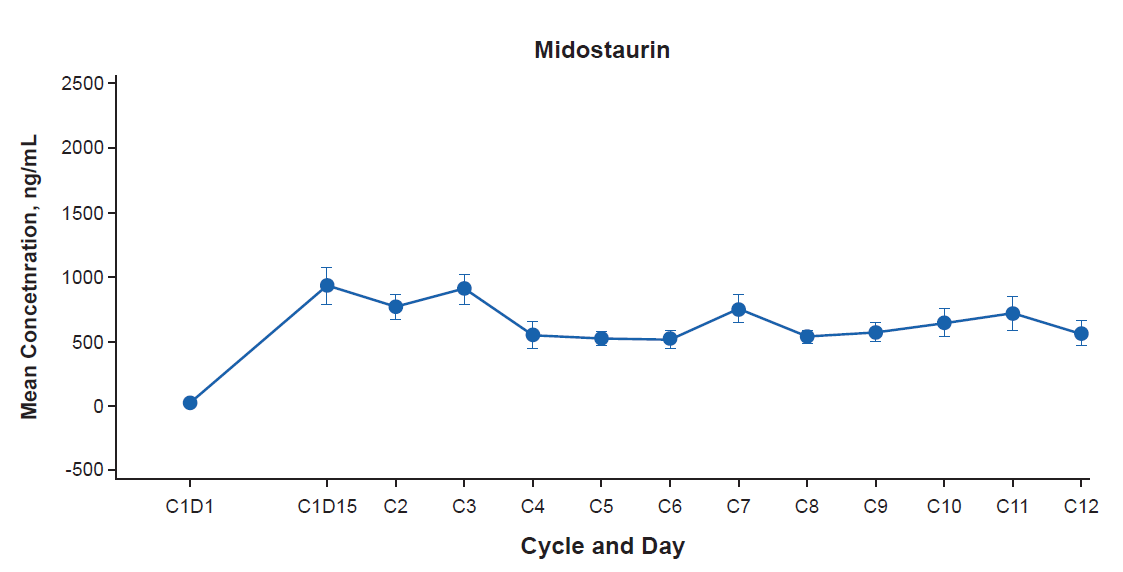


**B**

**
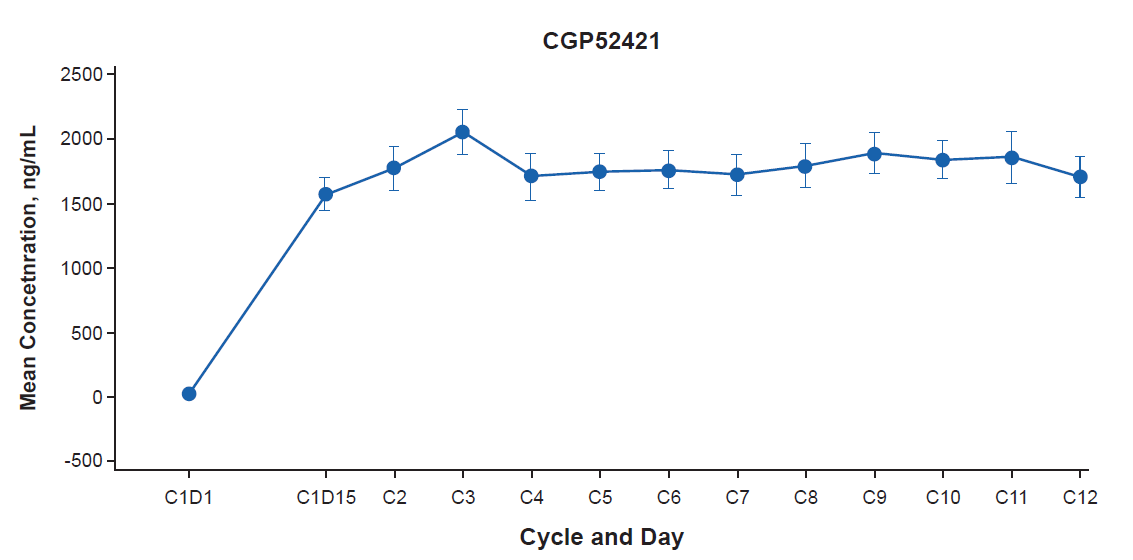
**

**C**

**
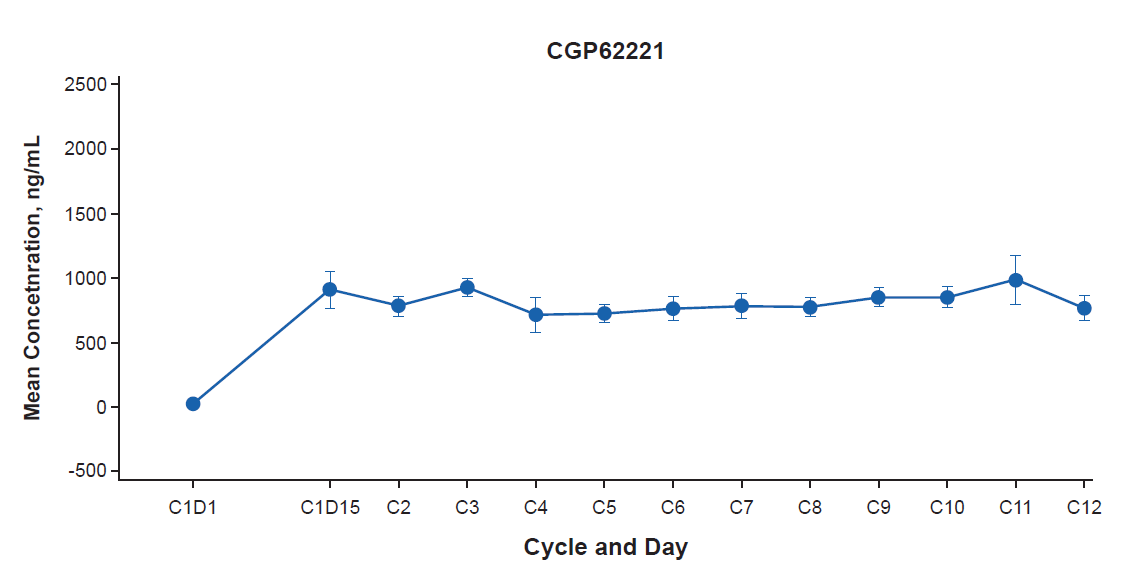
**

**Figure S3.** Among 56 patients who reached cycle 3 day 1 (C3D1) of therapy, 28 had been randomized to the midostaurin + SOC arm. In an exploratory biomarker analysis to evaluate the correlation between FLT3 inhibitory levels and clinical outcomes, patients were stratified according to P-FLT3 levels above or below the median (70%). Among 14 patients who had high levels of FLT3 inhibition (P-FLT3 <70%), 10 completed 12 cycles of therapy. Individual patient P-FLT3 levels ranged from 20% to 69% from baseline. Among the remaining patients who had low levels of FLT3 inhibition (P-FLT3 >70%), 6 patients completed 12 cycles of therapy. C, cycle; D, day; FLT3, fms-like tyrosine kinase 3; P-FLT3, phosphorylated FLT3; SOC, standard of care. ^a^ P-FLT3 levels were not measured (n=8). ^b^ Patient not on drug on C3D1 (n=6).


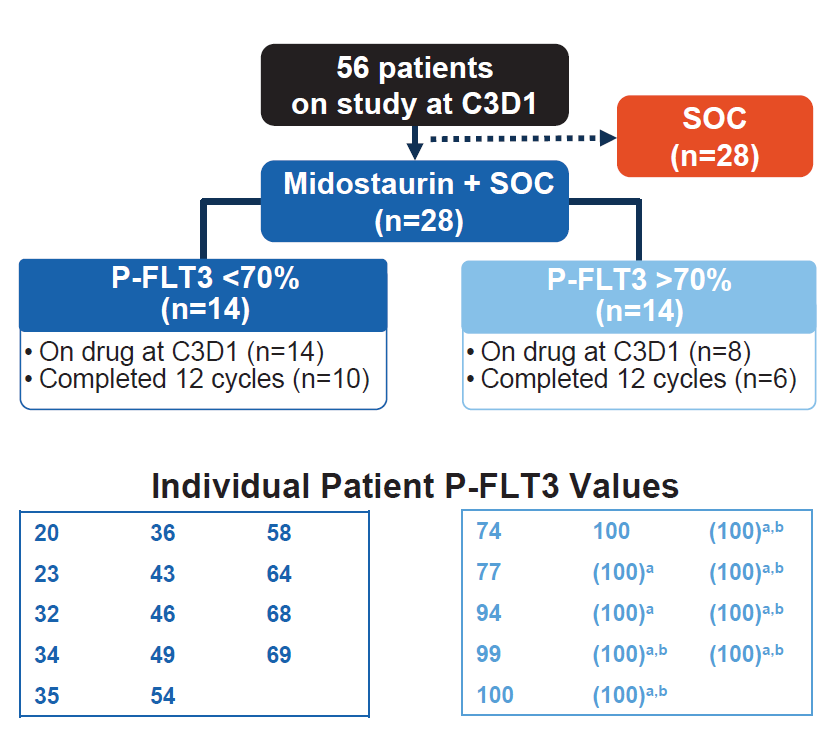


**Figure S4.** Extent of organ involvement with GVHD.^a^ GI, gastrointestinal; GVHD, graft-vs-host disease; M, midostaurin; SOC, standard of care. ^a^ Of patients whose GVHD was graded.


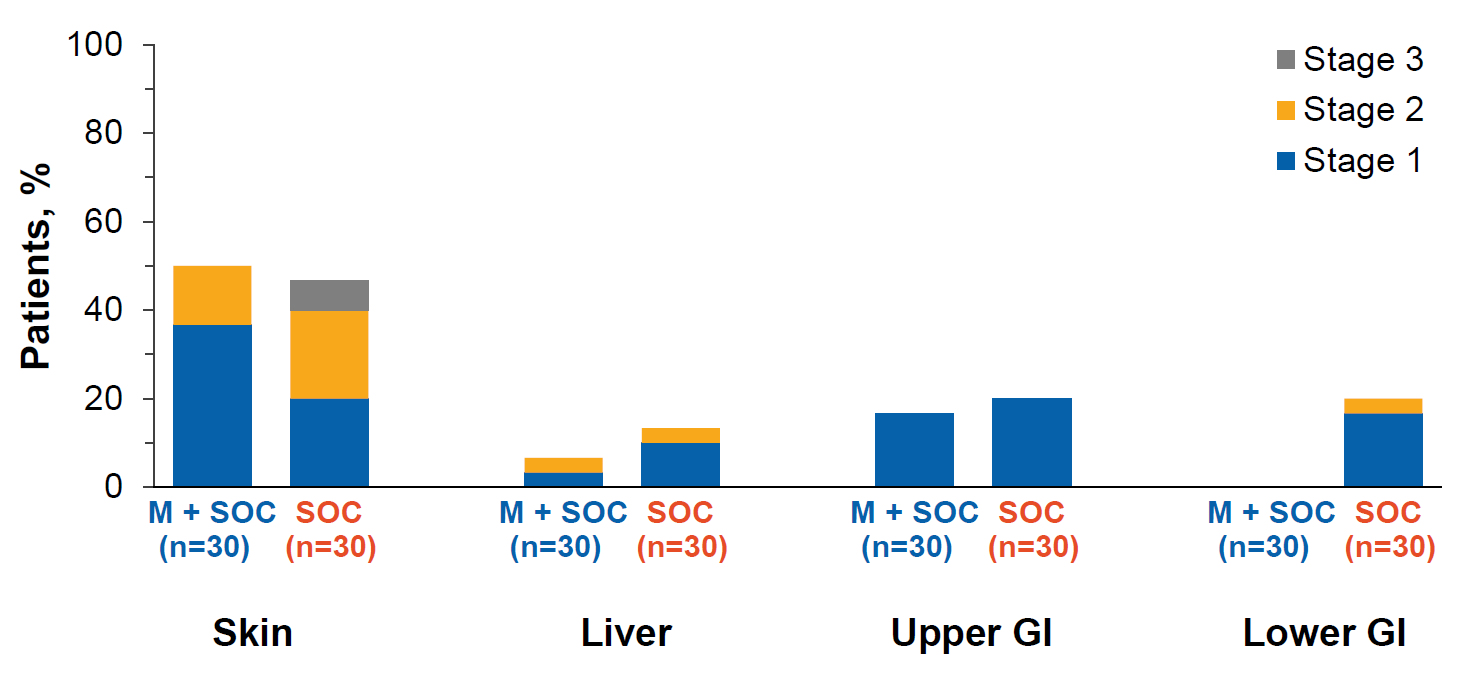


**Supplemental References**

1. Stone RM, Mandrekar SJ, Sanford BL, Laumann K, Geyer S, Bloomfield CD *et al.* Midostaurin plus chemotherapy for acute myeloid leukemia with a FLT3 mutation. *N Engl J Med* 2017; **377**(5)**:** 454-464. e-pub ahead of print 2017/06/24; doi: 10.1056/NEJMoa1614359

2. Perl AE, Altman JK, Cortes J, Smith C, Litzow M, Baer MR *et al.* Selective inhibition of FLT3 by gilteritinib in relapsed or refractory acute myeloid leukaemia: a multicentre, first-in-human, open-label, phase 1-2 study. *Lancet Oncol* 2017; **18**(8)**:** 1061-1075. e-pub ahead of print 2017/06/25; doi: 10.1016/S1470-2045(17)30416-3

3. Knapper S, Russell N, Gilkes A, Hills RK, Gale RE, Cavenagh JD *et al.* A randomized assessment of adding the kinase inhibitor lestaurtinib to first-line chemotherapy for FLT3-mutated AML. *Blood* 2017; **129**(9)**:** 1143-1154. e-pub ahead of print 2016/11/23; doi: 10.1182/blood-2016-07-730648

4. Levis M, Brown P, Smith BD, Stine A, Pham R, Stone R *et al.* Plasma inhibitory activity (PIA): a pharmacodynamic assay reveals insights into the basis for cytotoxic response to FLT3 inhibitors. *Blood* 2006; **108**(10)**:** 3477-3483. e-pub ahead of print 2006/07/22; doi: 10.1182/blood-2006-04-015743

5. Sato T, Yang X, Knapper S, White P, Smith BD, Galkin S *et al.* FLT3 ligand impedes the efficacy of FLT3 inhibitors in vitro and in vivo. *Blood* 2011; **117**(12)**:** 3286-3293. e-pub ahead of print 2011/01/26; doi: 10.1182/blood-2010-01-266742
